# The effect of desflurane, isoflurane and sevoflurane on the hemoglobin oxygen dissociation curve in human blood samples

**DOI:** 10.1038/s41598-022-17789-6

**Published:** 2022-08-10

**Authors:** Marco Ronzani, Simon Woyke, Norbert Mair, Hannes Gatterer, Herbert Oberacher, David Plunser, Thomas Haller, Mathias Ströhle, Christopher Rugg

**Affiliations:** 1 Department of Anesthesiology and Critical Care Medicine, Medical University of Innsbruck, Anichstraße 35, 6020 Innsbruck, Austria; 2 Institute of Physiology, Medical University of Innsbruck, Innsbruck, Austria; 3 Institute of Mountain Emergency Medicine, Eurac Research, Bolzano, Italy; 4 Institute of Legal Medicine and Core Facility Metabolomics, Medical University of Innsbruck, Innsbruck, Austria

**Keywords:** Physiology, Medical research, Molecular medicine

## Abstract

Desflurane, isoflurane and sevoflurane, three halogenated ethers, are commonly used inhaled anesthetics, both in the operating room and in the intensive care unit (ICU). The potency and dosage of these drugs is expressed by the MAC value (minimum alveolar concentration). Their interaction with hemoglobin and its affinity for oxygen, best described by the oxygen dissociation curve (ODC), has already been investigated, with conflicting results. Altered by many factors, the ODC can be shifted to the left or to the right, therefore increasing or decreasing hemoglobin oxygen (Hb-O_2_) affinity. In venous blood samples of 22 healthy participants (11 female, 11 male) ODC were measured with a high-throughput method in vitro. Blood samples were either exposed to control or to three different concentrations of desflurane, isoflurane or sevoflurane prior to and during measurements (low, medium and high corresponding to MAC 0.5, MAC 1.0 and MAC 2.0). With increasing concentrations from control to medium, desflurane and isoflurane significantly decreased Hb-O_2_ affinity by shifting the ODC to the right (p = 0.016 and p < 0.001) but sevoflurane showed no effects. When further increasing concentrations from medium to high, all three inhaled anesthetics shifted the ODC back to the left (p < 0.001). Comparing only controls to high concentrations, a significant increase in Hb-O_2_ affinity for desflurane (p = 0.005) and sevoflurane (p < 0.001) was detected. Our study shows a varying effect at different doses of inhaled anesthetics on Hb-O_2_ affinity. While the underlying mechanisms remain unclear, these results show an effect which needs to be further investigated to determine if patients undergoing anesthesia may potentially benefit or get disadvantage from this slightly increased (e.g. impaired pulmonary oxygen uptake), or decreased Hb-O_2_ affinity (e.g. arterial vascular disease).

**Trial registration:** This study is registered with clinicaltrials.gov (NCT04612270).

## Introduction

In mammals, the essential role of transporting oxygen(O_2_) from the lungs to the tissues is provided by the molecule hemoglobin (Hb)^1^. The interaction between hemoglobin and oxygen is represented by the oxygen dissociation curve (ODC), which was first reported in the early 1900s by Bohr, who demonstrated a reduced hemoglobin oxygen (Hb-O_2_) affinity in acidic conditions and described the sigmoid shape of the curve^2^. The ODC is best described by two main parameters: the p50, representing the partial pressure of O_2_ at 50% Hb-O_2_ saturation, and the Hill Coefficient (HC), representing the maximum steepness of the curve in the logarithmic Hill plot^3^. Many agents and conditions have the capability to modify the interaction between hemoglobin and O_2_, indicated by a change in the ODC. In general, a shift to the right (increase in p50) indicates a decrease, whereas a shift to the left indicates an increase in Hb-O_2_ affinity^1^.

Inhaled anesthetics play a central role in modern anesthesia. Desflurane, isoflurane and sevoflurane—all halogenated ethers—are the most commonly used inhaled anesthetics in modern operation theaters and are often used in combination with intravenous anesthetics and analgesics for anesthesia maintenance^4^. In intensive care medicine inhaled anesthetics are used for sedation, particularly in the context of distinct tolerance or tachyphylaxis to intravenous sedatives, severe bronchospasms and epilepsy refractory to regular treatment or as anesthetic preconditioning^5^.

In the clinical setting the potency and dosage of inhaled anesthetics are often described by the Minimum Alveolar Concentration (MAC), which expresses the concentration at sea level where 50% of the volunteers did not show purposeful movement after a surgical stimulus^6^. The MAC value is mainly influenced by age, but also by temperature, barometric pressure and blood sodium levels^6^. For 18–30 year-olds, MAC 1 values for desflurane, isoflurane and sevoflurane are 7.25%, 1.3% and 2.4%^7^, respectively.

The choice between these drugs depends on several factors, amongst them the interaction with the cardiovascular system. While desflurane and sevoflurane show the strongest negative inotropic effects, desflurane and isoflurane can cause an increase in heart rate. With regard to the respiratory system, all three anesthetics cause a bronchodilation, while desflurane and isoflurane can trigger laryngeal spasms and are therefore considered unfeasible for induction of anesthesia^8^.

The interaction of inhaled anesthetics with Hb-O_2_ affinity was already investigated in the ‘70s and ‘80s of the twentieth century, where the studies conducted by Smith et al. were able to show that halothane, enflurane and nitrous oxide shift the ODC to the right, thereby decreasing Hb-O_2_ affinity^9^. On the other hand, Lanza et al. reported no change in Hb-O_2_ affinity for halothane and enflurane, but confirmed the abovementioned effects for nitrous oxide^10^. Furthermore, Kambam et al. also showed a right shift of the ODC not only for nitrous oxide but also for isoflurane^11,12^, while for sevoflurane no effects were found^13^. Overall, the effects of various inhaled anesthetics on the ODC and the influence of differing dosages is not well established and for some anesthetics even contradictory. Therefore, the influence on Hb-O_2_ affinity cannot be taken into consideration by clinicians up to date.

In this study, we aimed to investigate the effect at different dosage of the commonly used inhaled anesthetics desflurane, isoflurane and sevoflurane on Hb-O_2_ affinity in whole blood samples using a new in vitro method for ODC determination.

## Materials and methods

Venous blood samples were drawn with a minimum period of stasis from 11 female and 11 male healthy volunteers aged between 18 and 40 years. All subjects were nonsmokers, not pregnant or breastfeeding, had no known hemoglobinopathy, or recent history of illness, trauma, recent surgery, blood loss or multi-day trips to high altitude (> 3000 m). Immediately after blood sampling, the heparinized samples were stored on ice, while blood gas analysis (ABL 800 flex, Radiometer) was performed from aliquots. Another aliquot was separated and stored at − 80 °C for the quantification of 2,3-bisphosphoglycerate (2,3-BPG) and adenosine triphosphate (ATP) concentrations with a validated liquid chromatography–tandem mass spectrometry method described before^14^.

The in-vitro ODC measurements were performed with a high-throughput method for the recording of ODCs^15^. In the four channel ODC plate, blood samples and an internal hemoglobin standard solution^15^ were exposed to desflurane, isoflurane, and sevoflurane side by side with a control, exposed to anesthetic-free standard gas mixes only^15^. In three runs, samples were exposed to low, medium and high doses of inhaled anesthetics, corresponding to a MAC of 0.5, 1.0 and 2.0 when referring to the population present in this study (18–40 years, female and male; Table 1)^7^. Gas concentrations were measured at the end of the gas flow-through system with a multi-gas module and an intensive care monitor (Mindray Benevision with Multigas Module, Mindray Bio-Medical Electronics Co., Ltd, China).

**Table 1:** Concentrations of inhaled anesthetics based on MAC values adapted from Thiel & Roewer’s “Anästhesiologische Pharmakotherapie” used in this study^7^.

	MAC 0.5 (%)	MAC 1.0 (%)	MAC 2.0 (%)
Desflurane	3.75	7.5	15
Isoflurane	0.75	1.5	3
Sevoflurane	1.25	2.5	5

*MAC* minimal alveolar concentration.

Inhaled anesthetics were vaporized and volumetrically added to the oxygen containing and oxygen free gas mixes used for the in vitro method. Prior to the study, stability of inhaled anesthetics in the gas sampling bags was confirmed by preparation of a defined gas mixture and repeated measurements over 24 h using the above-mentioned gas monitor. Additionally, interference of the inhaled anesthetics with absorption measurements was excluded in advance. Triplicate measurements were performed for every concentration, substance and blood sample. The thermostatic experimental setup was set to 37 °C, all gas mixtures contained 40 mmHg PCO_2_.

Excel (Microsoft 2016) was used for curve fittings, P50 and HC calculations. Statistical analysis was performed using R (v4.0.2, R Core Team, www.R-project.org) and RStudio (v1.2.5001, RStudio Inc., Boston, MA, USA). Due to non-normal distribution of the small sample size, non-parametric tests were applied. ANOVA type statistics modified for non-parametric longitudinal data (R package: nparLD) was utilized to analyze the effects of different inhaled concentrations of anesthetics with regard to controls^16^. A p < 0.05 was considered significant. Data are presented as median and interquartile range (IQR; first and third quartile).

### Ethical approval

This study was approved by the ethics committee of the Medical University of Innsbruck (vote nr. 1265/2020) and is registered with clinicaltrials.gov (NCT04612270, 02/11/2020). The authors confirm that all experiments were performed in accordance with relevant guidelines and regulations. Written informed consent was given by all subjects.

## Results

A total of 22 subjects (11 female; 11 male) were included in the study. General demographics and baseline characteristics are presented in Table 2. Particularly hemoglobin concentration and hematocrit were within normal ranges for this study population. The pCO_2_ values in our venous whole blood samples were slightly elevated and pH on the lower end of the normal range. Baseline values used as control for p50 and HC were 27.9 (26.3–29.4) mmHg and 2.9 (2.7–3.0), respectively (Table 2). ODCs of all subjects for the three anesthetics at low, medium and high concentration are shown in Supplementary Material.

**Table 2:** Baseline characteristics of the study population (median and interquartile range, IQR).

	Median	IQR (Q1–Q3)
Age (y)	29.0	28.0–30.0
Hemoglobin (g/dl)	14.4	13.4–15.0
Hematocrit (%)	44.1	41.3–46.0
pCO_2_ (mmHg)	44.3	40.4–52.9
pH	7.36	7.33–7.38
P50 (mmHg)	27.9	26.3–29.4
Hill coefficient	2.9	2.7–3.0
2,3 BPG (µmol/gHb)	19.9	18.6–22.4
ATP (µmol/gHb)	5.0	4.7–5.5

*pCO*_*2*_ carbon dioxide partial pressure in blood, *p50* oxygen partial pressure at 50% saturation,* 2,3 BPG* 2,3-bisphospholycerate, *ATP* adenosine triphosphate.

Sex specific analysis revealed no difference between sexes therefore results of the combined data set is provided.

**Figure 1:**
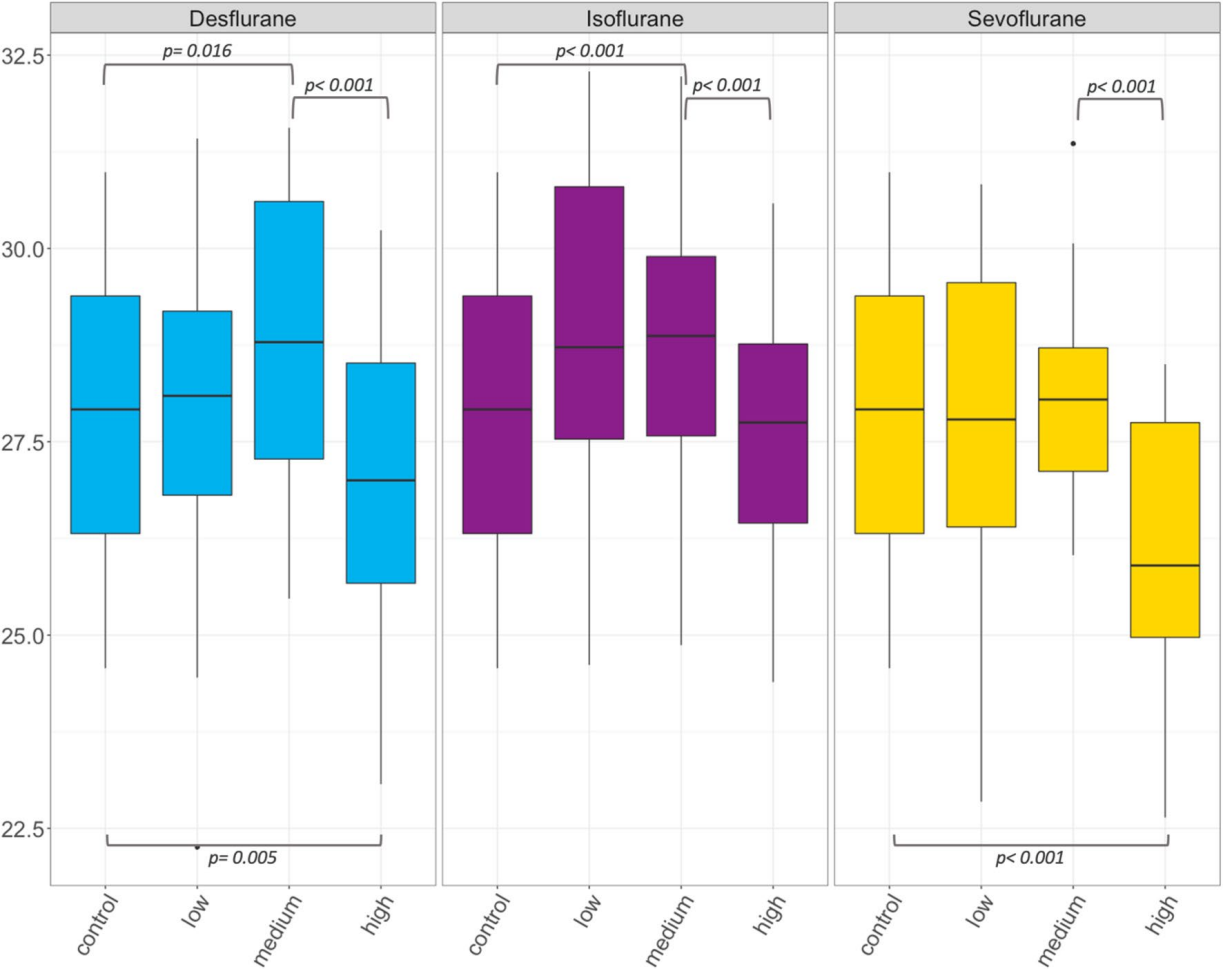
Box plots of p50 (in mmHg) for desflurane, isoflurane and sevoflurane at low (corresponding to MAC 0.5), medium (MAC 1.0) and high (MAC 2.0) concentration. Significant differences are indicated by brackets and p values are reported. P50 is oxygen partial pressure at 50% saturation.

**Figure 2:**
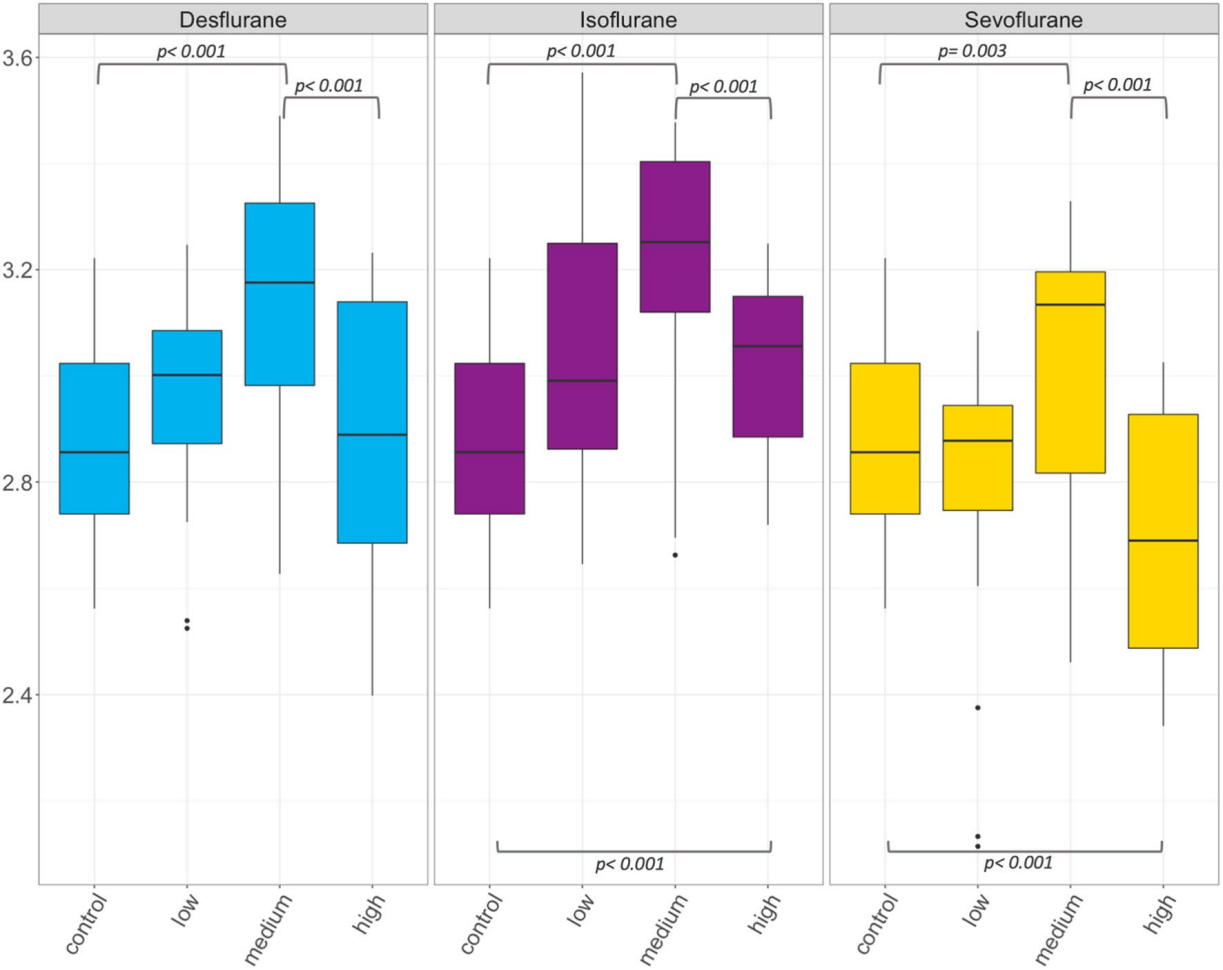
Box plots of HC for desflurane, isoflurane and sevoflurane at low (corresponding to MAC 0.5), medium (MAC 1.0) and high (MAC 2.0) concentration. Significant differences are indicated by brackets and p values are reported. HC is Hill coefficient.

Overall, a significant, unspecific effect of the examined volatile anesthetics on p50 and the HC was found (p < 0.001; Table 3; Figs. 1, 2). In detail, with increasing anesthetic gas concentrations from control over low to medium concentrations, a significant increase in p50 was shown for desflurane (p = 0.016) and isoflurane (p < 0.001) but not for sevoflurane (p = 0.613; Table 3; Fig. 1). Subsequently, desflurane, isoflurane and sevoflurane showed a significant decrease in p50 when further increasing the concentration from medium to high concentrations (p < 0.001; Fig. 1). Comparing controls to high concentrations only, a significant decrease in p50 was shown for desflurane (p = 0.005) and sevoflurane (p < 0.001), but not for isoflurane (p = 0.542).

**Table 3:** P50 and HC (median and interquartile range, IQR) of controls and three inhaled anesthetics at low, medium and high concentration.

	Desflurane median (IQR)		Isoflurane median (IQR)		Sevoflurane median (IQR)	
	P50 (mmHg)	HC	P50 (mmHg)	HC	P50 (mmHg)	HC
Control	27.9 (26.3–29.4)	2.9 (2.7–3.0)	27.9 (26.3–29.4)	2.9 (2.7–3.0)	27.9 (26.3–29.4)	2.9 (2.7–3.0)
Low concentration	28.1 (26.8–29.2)	3.0 (2.9–3.1)	28.7 (27.5–30.8)	3.0 (2.9–3.3)	27.8 (26.4–29.6)	2.9 (2.7–2.9)
Medium concentration	28.8 (27.3–30.6)	3.2 (3.0–3.3)	28.9 (27.6–29.9)	3.3 (3.1–3.4)	28.1 (27.1–28-7)	3.1 (2.8–3.2)
High concentration	27.0 (25.7–28.5)	2.9 (2.7–3.1)	27.8 (26.5–28.8)	3.1 (2.9–3.2)	25.9 (25.0–27.8)	2.7 (2.5–2.9)

*P50* oxygen partial pressure at 50% saturation, *HC* the Hill coefficient.

With regard to the HC, desflurane (p < 0.001), isoflurane (p < 0.001) and sevoflurane (p = 0.003) led to a significant increase while progressing from control over low to medium concentrations (Fig. 2). Further increasing gas concentrations from medium to high significantly decreased the HC again for all volatiles (p < 0.001). Compared to controls only, the HC in high concentrations did not differ for desflurane (p = 0.437), increased for isoflurane (p < 0.001) and decreased for sevoflurane (p < 0.001).

## Discussion

In this study, significant effects of desflurane, isoflurane and sevoflurane on p50, HC and hence Hb-O_2_ affinity in human whole blood samples are shown. With varying effects at different doses, p50 and HC increased with desflurane and isoflurane from controls to medium concentrations but subsequently decreased substantially when further increasing the applied concentrations to a high level. Sevoflurane however, showed no effect on p50 up to medium concentrations followed by a significant decrease when applying high concentrations. Compared to controls only, the total effect of high concentrations was (1) a significant decrease of p50 for desflurane and sevoflurane but non-significant differences for isoflurane and (2) no difference in the HC for desflurane, an increase of the HC for isoflurane and a decrease of the HC for sevoflurane. The described effects translate into an initial right shift of the ODC and therefore a decrease in Hb-O_2_ affinity for desflurane and isoflurane up to medium concentrations, followed by a subsequent increase again when further increasing the dose. Although unaffected by low to medium concentrations, sevoflurane was able to increase Hb-O_2_ affinity the most by decreasing p50 when applying high concentrations.

These findings are partially in line with the results of Kambam et al.^11,12^, who showed an ODC shift to the right for isoflurane and nitrous oxide. Furthermore, in their studies, no influence of sevoflurane on Hb-O_2_ affinity was detected. Noteworthy, the applied concentrations approximately corresponded to our low to medium concentrations where also no effects on Hb-O_2_ affinity was shown^13^. In contrast to our findings, Wade et al. reported that isoflurane at a concentration of 2%, corresponding to a MAC between 1 and 2, increased the oxygen affinity of sickle hemoglobin, shifting the ODC to the left^17^. Of course, sickle cell hemoglobin per se exhibits decreased oxygen affinity potentially explaining discrepancies when comparing results to healthy adults.

The underlying mechanisms leading to the reported modification of Hb-O_2_ affinity by inhaled anesthetics are unclear. An interaction with several receptors, modifying the electrolyte balance and, as a consequence, intracellular pH in red blood cells seems possible: Altikat et al. showed that the activity of glucose-6-phosphate dehydrogenase (G6PD) in red blood cells was inhibited by sevoflurane and isoflurane, but not by halothane. Thus, the production of NAPDH was reduced, hence exposing the red blood cell to reactive oxygen species^18^. Fomitcheva et al. showed that isoflurane in clinically relevant concentrations inhibits the activity of plasma membrane Ca^2+^-transporting adenosine triphosphatase (PMCA), thus increasing intracellular Ca^2+^ concentrations^19^. This increase was linked to a right shift of the ODC and a lower affinity for oxygen. As a potential cause, red blood cell shrinkage, resulting in an increase in 2,3-DPG levels was ruled out as the right shift of the ODC was also observed in red blood cells where shrinkage was prevented. It was hypothesized that the effects are based on an intracellular pH change due to interactions of increased free Ca^2+^-concentrations with the protonation of hemoglobin^20,21^. Further increasing the concentration to a MAC of approximately 3 was also shown to inhibit several types of ATPase like the Na^+^-K^+^-ATPase or the Mg^2+^-ATPase^19^. This may partially explain the underlying mechanisms behind the opponent effects on Hb-O_2_ affinity when progressing the dose to rather high concentrations, as observed in our study. Moreover, possible genetic influences on drug effects at an individual level, in terms of pharmacogenetics, must be taken into account^22,23^.

Although the demonstrated shift in p50 might seem minor, clinical relevance cannot be excluded and needs to be further evaluated. Whether a shift of the ODC to the right or to the left may be beneficial is still under debate and certainly depends on the individual patient and his underlying disease^24^. Changes in pulmonary O_2_ absorption capacity may be counteracted by numerous factors at the peripheral tissue level. A left shift might be capable to increase the oxygen absorption in the lungs, but at the same time it may reduce oxygen delivery at the peripheral cellular level. On the other side, Woodson et al. showed that in rats, a left shifted ODC can raise the coronary and brain blood flow without increasing the workload of the heart—similar to anemia^25^. However, at the tissue level hypercapnia, acid metabolites, and even local hyperthermia might easily mitigate these effects. A right shift of the ODC on the other hand, can increase the amount of O_2_ released in the tissues, although a reduced absorption of oxygen in the lungs must be considered. In patients suffering from an oxygenation limitation, for example COVID-19 pneumonia or ARDS, a left shift of the ODC might be beneficial in improving the O_2_ uptake in the lungs^26^. On the other hand, in heart failure, peripheral vascular disease and other conditions where tissue oxygenation is at risk, a right shift of the ODC might mitigate tissue hypoxia.

An anesthetist must consider several parameters when choosing the optimal anesthetic for the given patient. Cardiovascular risk profile, obstructive pulmonary disease, type of surgery and required depth of anesthesia, just to name a few. Currently, inhaled anesthetics are standard care in general anesthesia^7^. Despite many common properties, each inhaled anesthetic has its own characteristics: desflurane seems to maintain cardiopulmonary function at best, sevoflurane outperforms in terms of adverse effects like airway irritation, and isoflurane shows a high blood-gas partition coefficient, thus minimalized consumption and faster induction and awakening time^4,8^.

The shown decrease in Hb-O_2_ affinity by desflurane and isoflurane in low to medium concentrations, increase in Hb-O_2_ affinity by desflurane and sevoflurane in high concentrations as well as absent influence on Hb-O_2_ affinity by sevoflurane in low to medium concentrations, needs to be further investigated since it is still unclear if the effects shown in this experimental setting may translate into clinically relevant effects.

### Limitations

Due to the in vitro design of our study, further studies are certainly necessary to confirm our result by investigating the impact of all three inhaled anesthetics in vivo. In addition, underlying pathophysiological mechanisms require further investigation.

## Conclusions

In low to medium concentrations in vitro, the use of isoflurane or desflurane is accompanied by a decrease in Hb-O_2_ affinity, where sevoflurane seems to have no effects. At high concentrations in vitro, desflurane and sevoflurane increase Hb-O_2_ affinity.

## Supplementary Information

Supplementary Information.

## Data Availability

The raw data supporting the conclusions of this article will be made available by the corresponding author upon reasonable request.
