# Supplementary material for: The effect of desflurane, isoflurane and sevoflurane on the hemoglobin oxygen dissociation curve in human blood samples
Source: Sci Rep. 2022 Aug 10;12:13633. doi: 10.1038/s41598-022-17789-6 (PMC9365211; doi:10.1038/s41598-022-17789-6)
Supplement: Supplementary file 1 — Supplementary Information. [file 41598_2022_17789_MOESM1_ESM.docx]

Supplementary Material to:

**“The effect of desflurane, isoflurane and sevoflurane on the hemoglobin oxygen dissociation curve in human blood samples”**


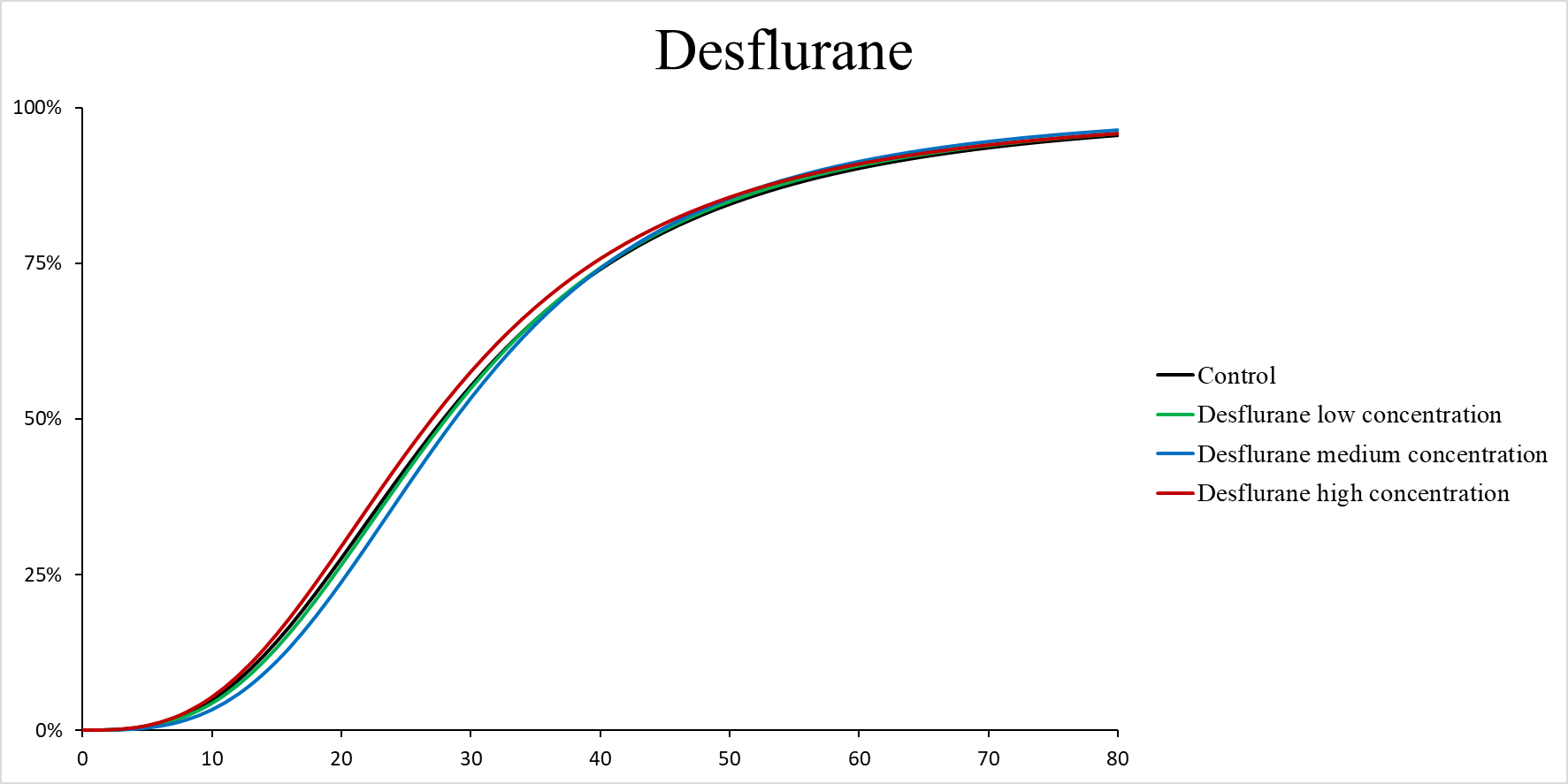


Fig S1: The varying effects of different concentrations of desflurane on the oxygen dissociation curve (ODC) are shown. ODCs are plotted based on median p50 and median HC of all subjects for each concentration. Y-axis is SO_2_ in percent and x-axis is PO_2_ in mmHg.


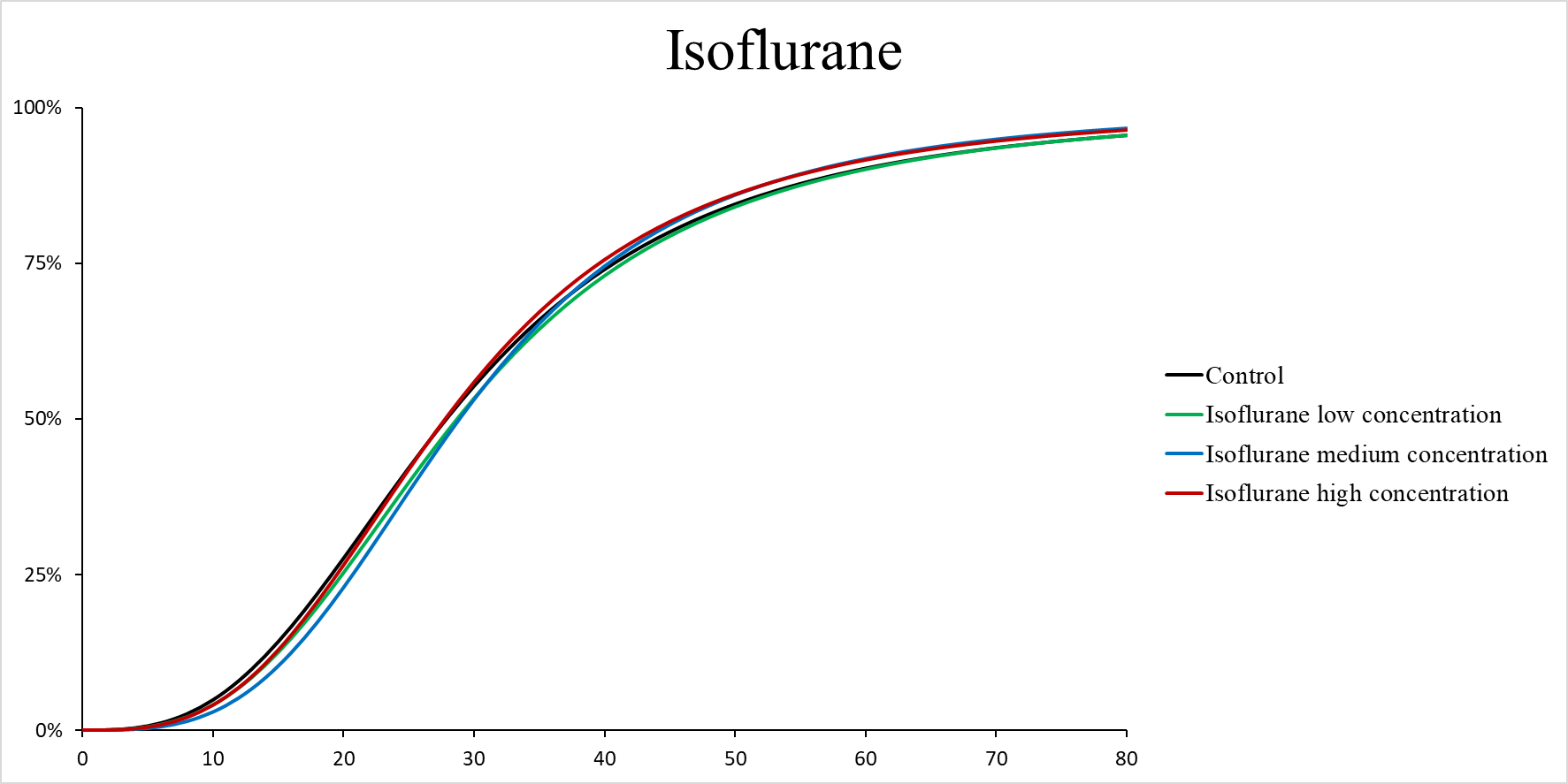


Fig S2: The varying effects of different concentrations of isoflurane on the ODC are shown. ODCs are plotted based on median p50 and median HC of all subjects for each concentration. Y-axis is SO_2_ in percent and x-axis is PO_2_ in mmHg.


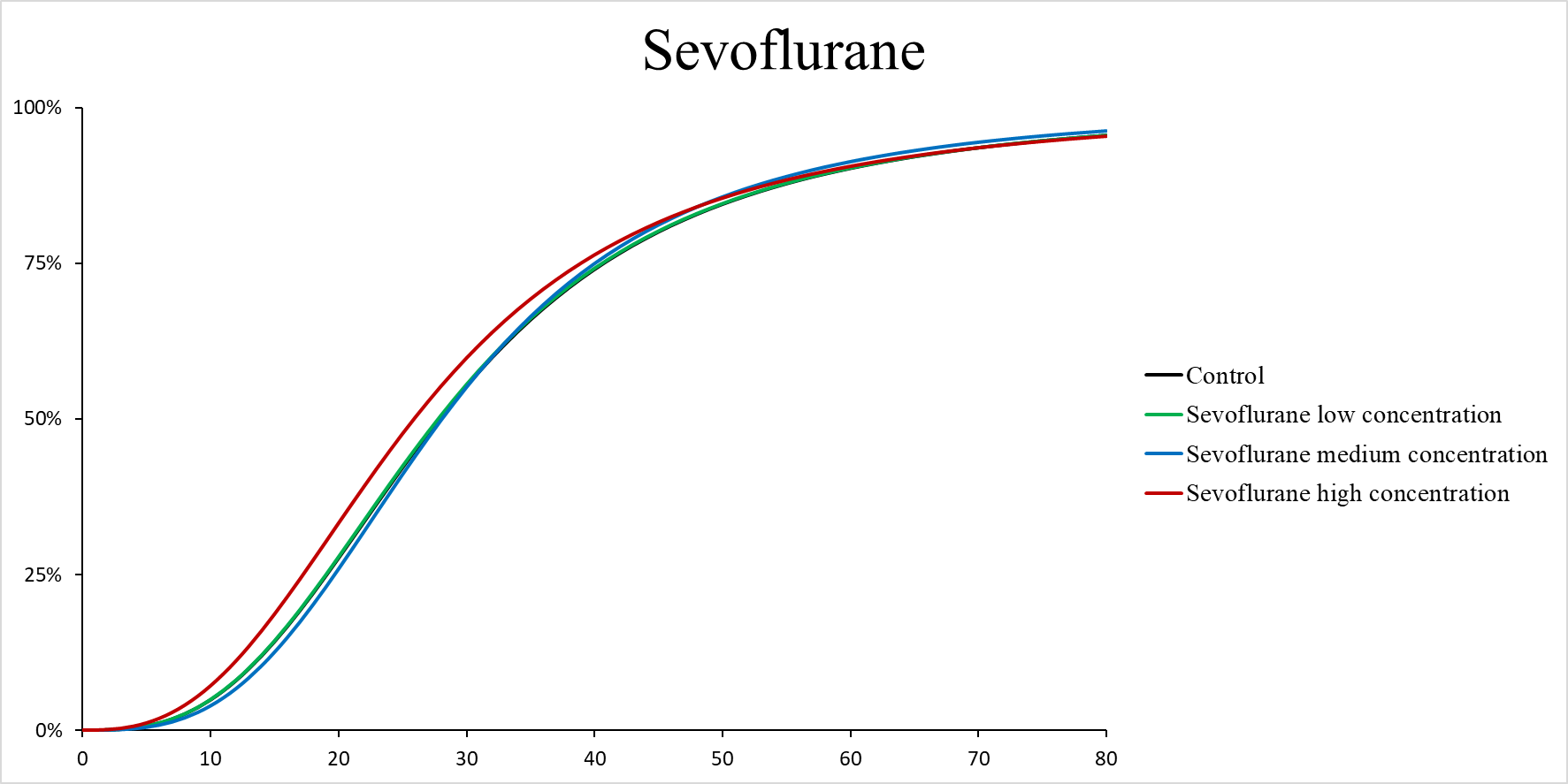


Fig S3: The varying effects of different concentrations of sevoflurane on the ODC are shown. ODCs are plotted based on median p50 and median HC of all subjects for each concentration. Y-axis is SO_2_ in percent and x-axis is PO_2_ in mmHg.


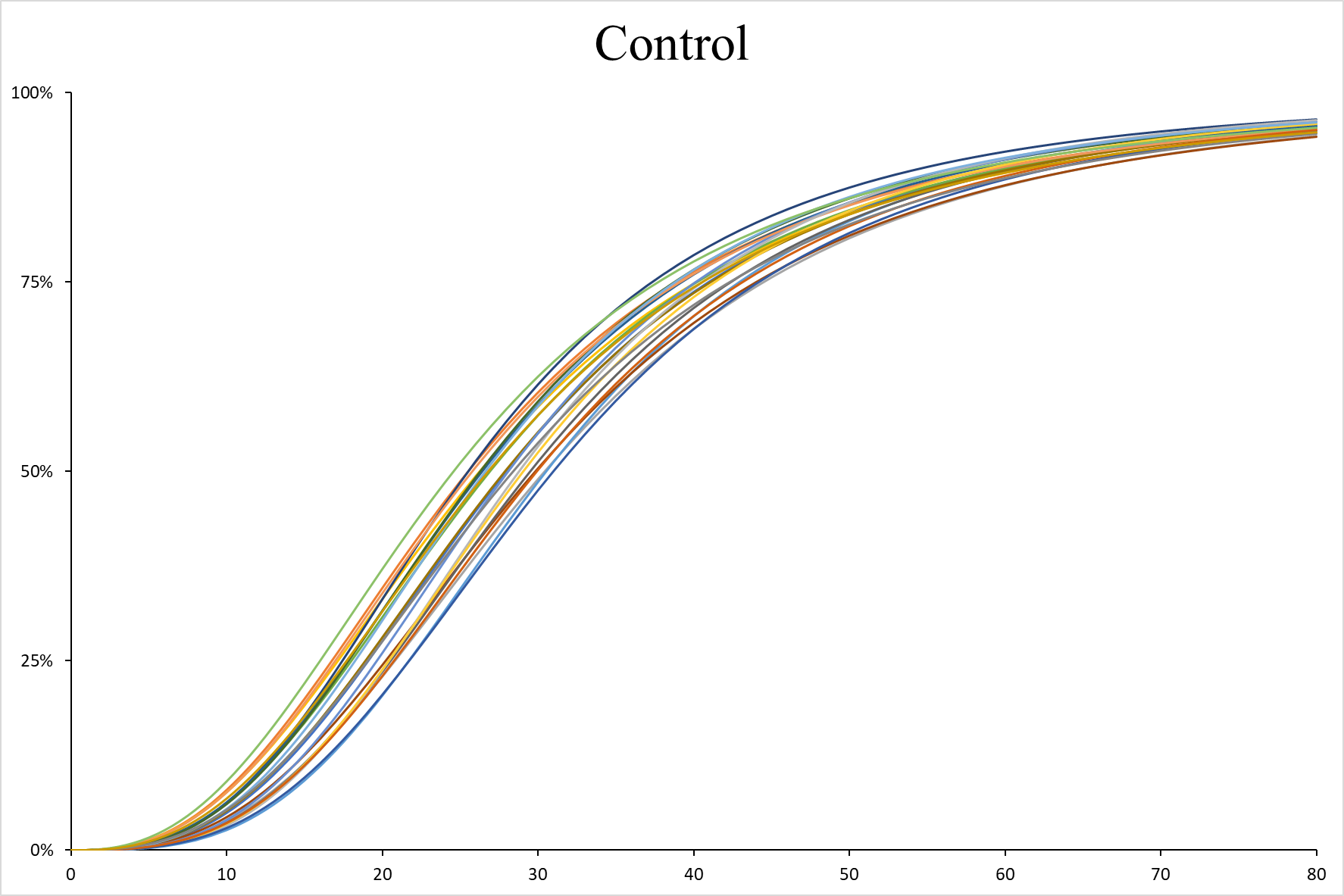


Fig S4: ODCs of all subjects (n = 22) are plotted for controls. Y-axis is SO_2_ in percent and x-axis is PO_2_ in mmHg.


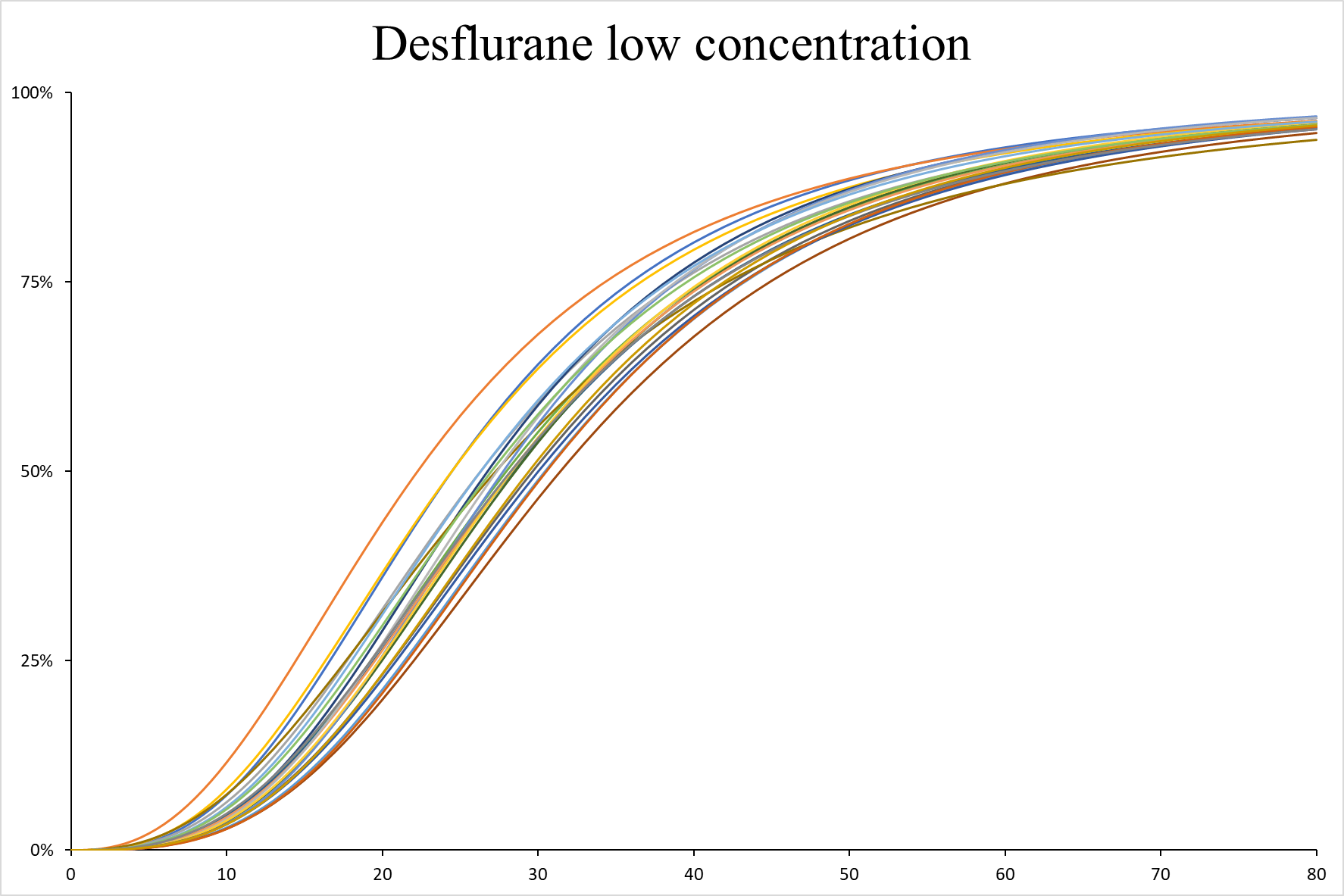


Fig S5: ODCs of all subjects (n = 22) are plotted for desflurane low concentration. Y-axis is SO_2_ in percent and x-axis is PO_2_ in mmHg.


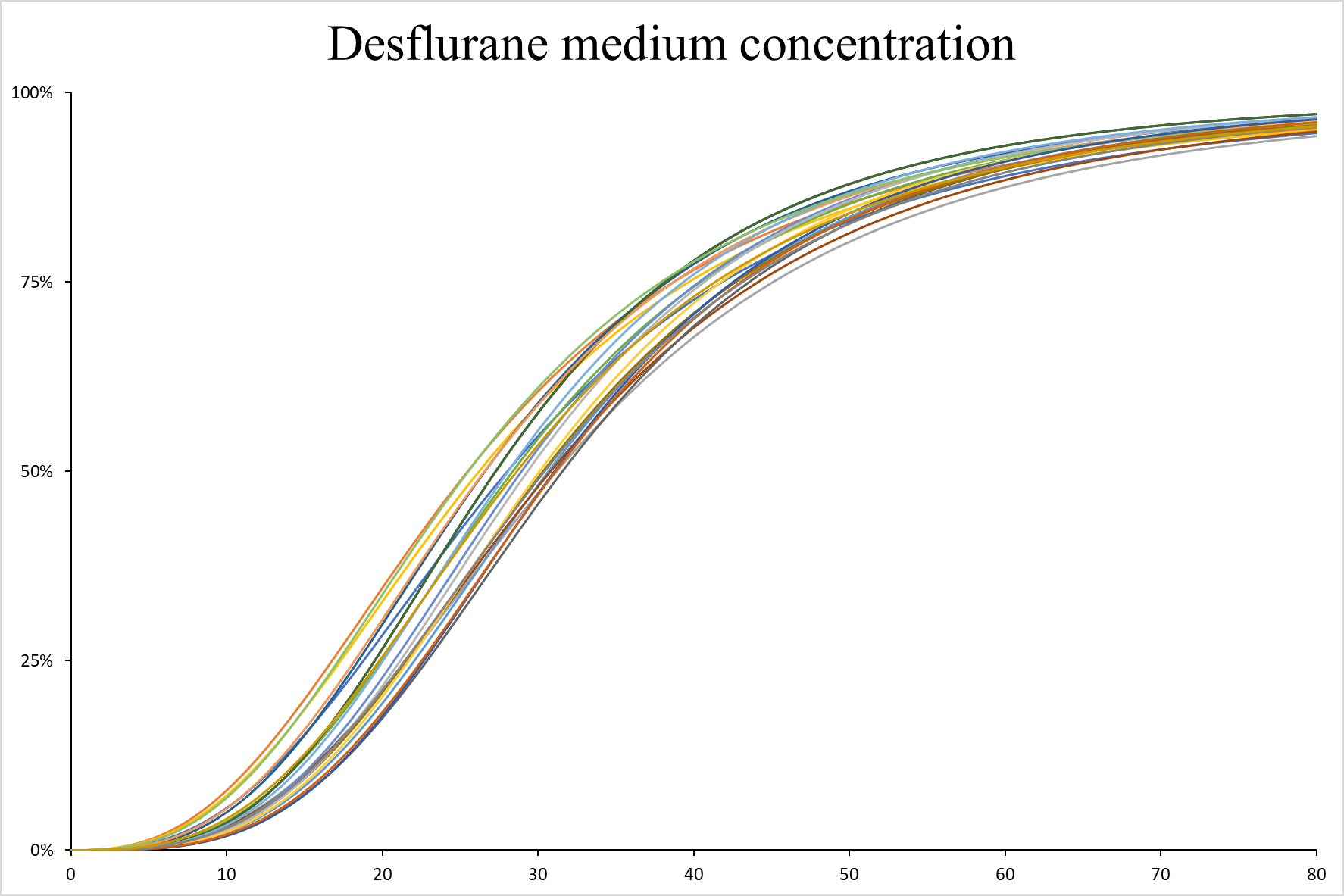


Fig S6: ODCs of all subjects (n = 22) are plotted for desflurane medium concentration. Y-axis is SO_2_ in percent and x-axis is PO_2_ in mmHg.


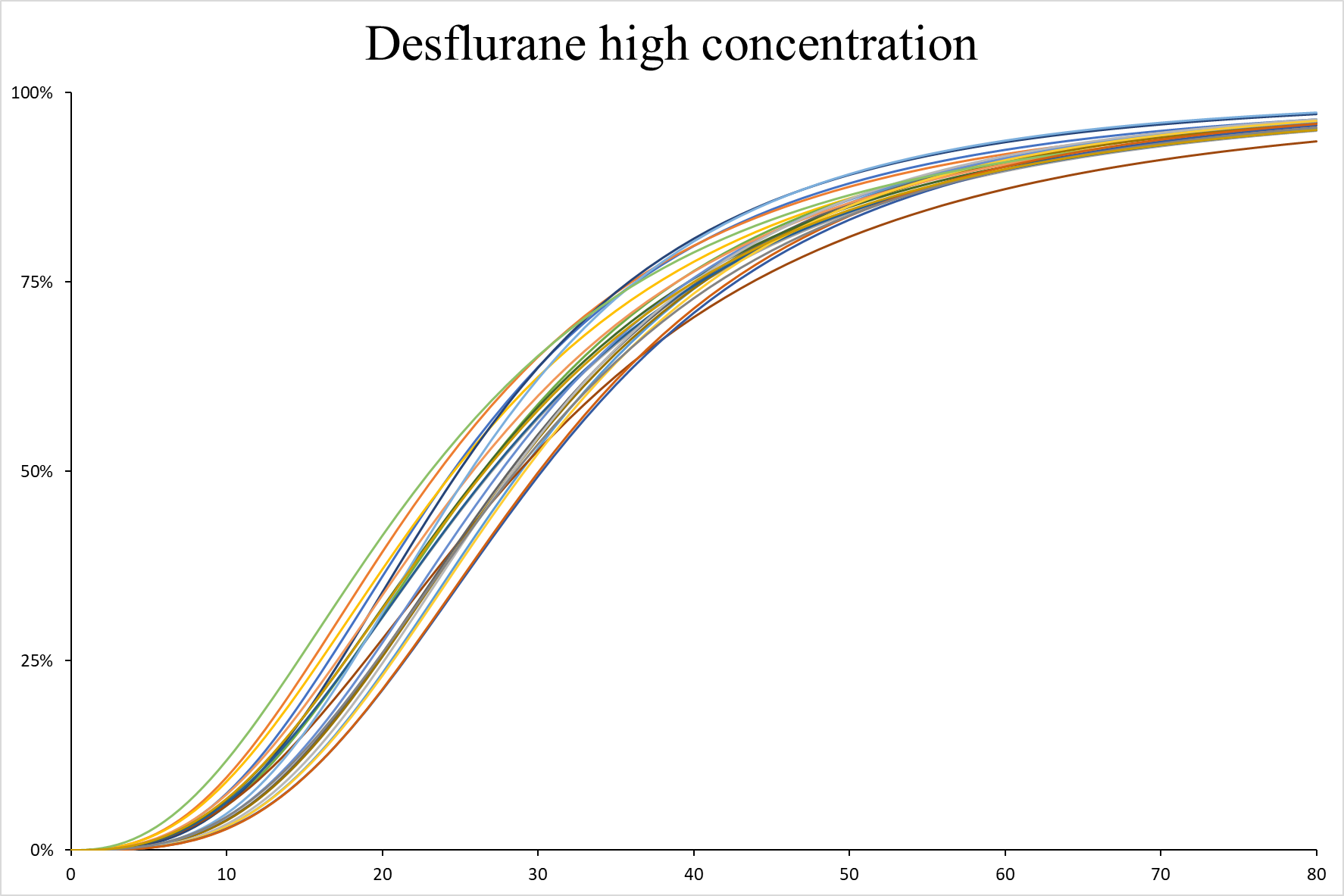


Fig S7: ODCs of all subjects (n = 22) are plotted for desflurane high concentration. Y-axis is SO_2_ in percent and x-axis is PO_2_ in mmHg.


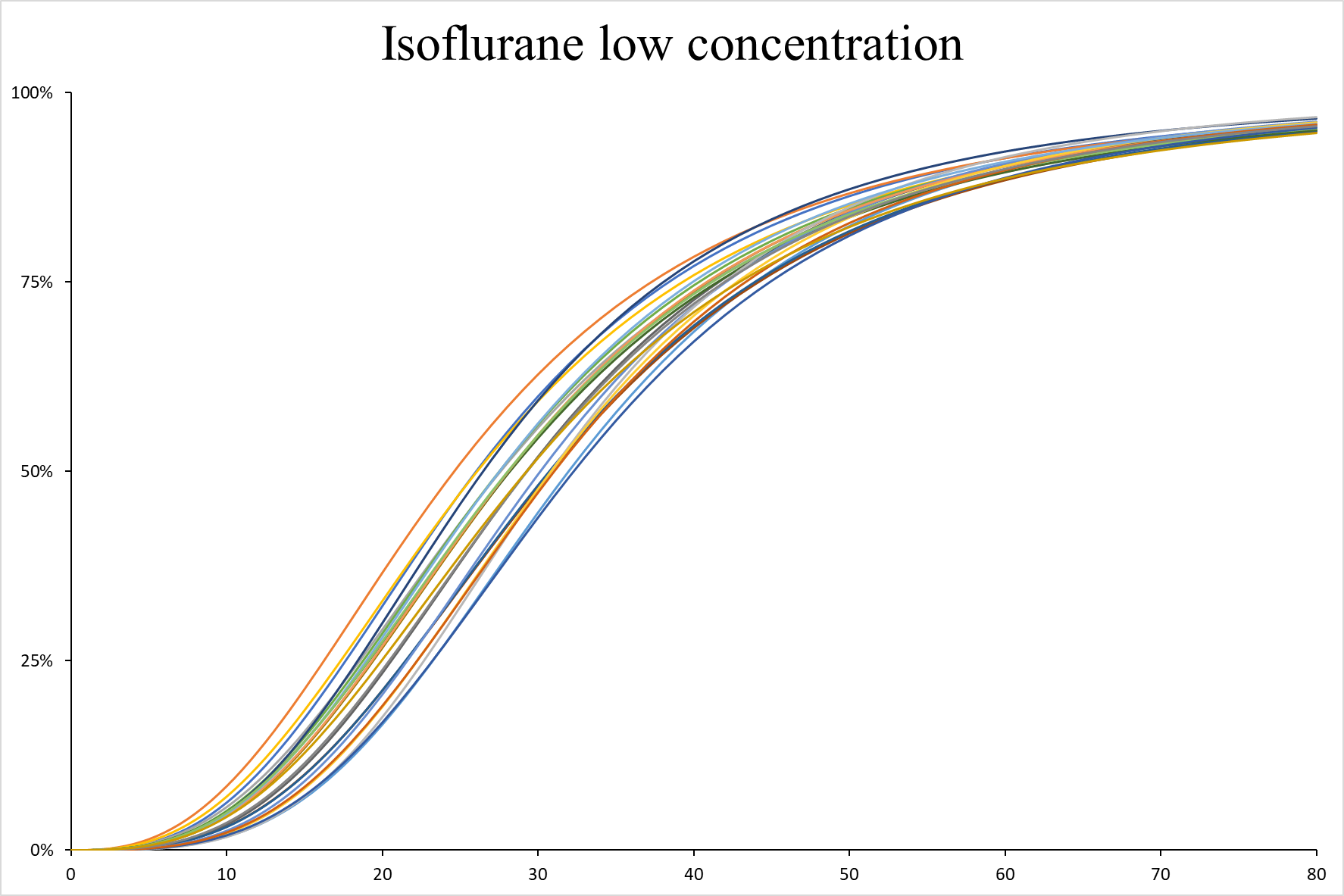


Fig S8: ODCs of all subjects (n = 22) are plotted for isoflurane low concentration. Y-axis is SO_2_ in percent and x-axis is PO_2_ in mmHg.


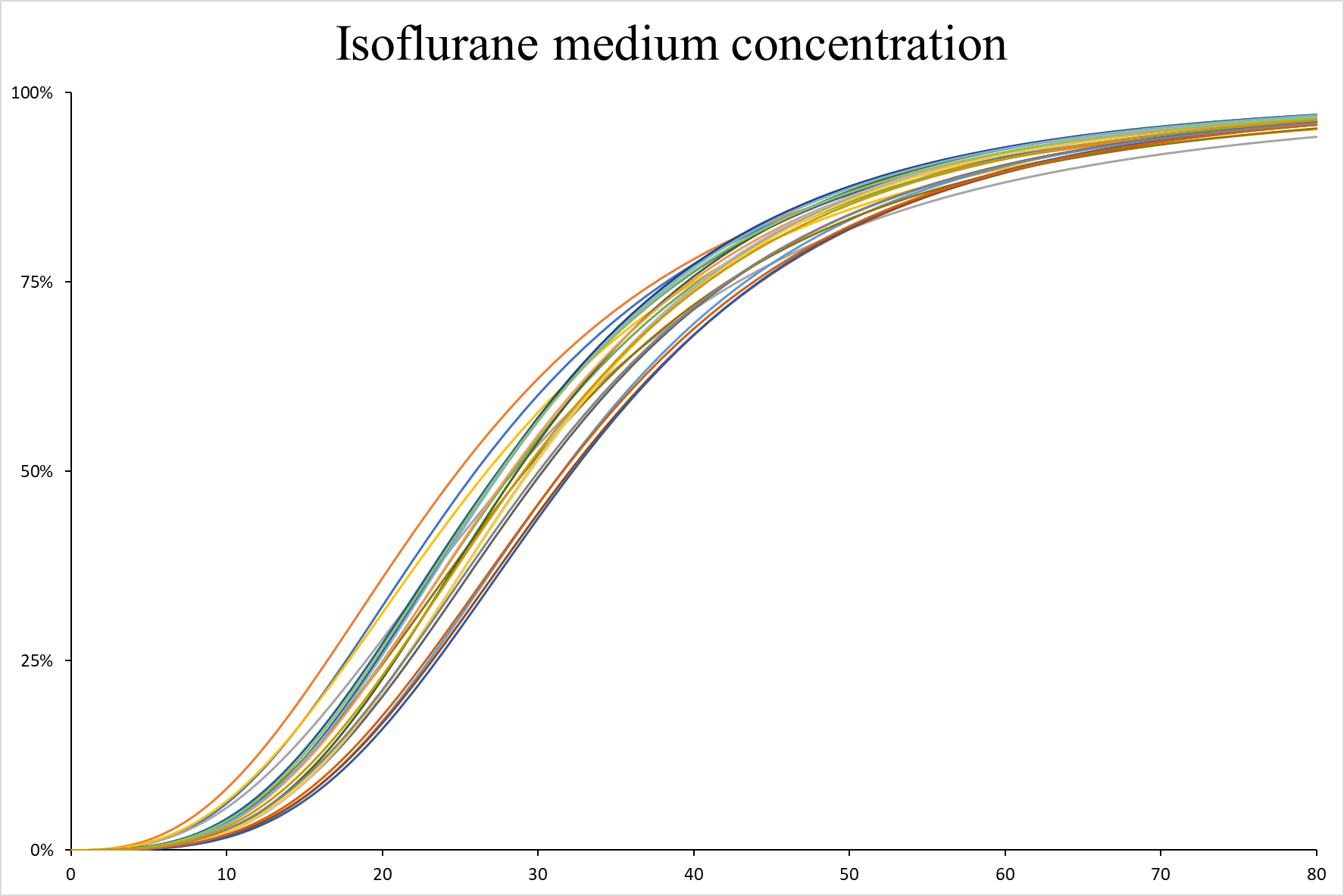


Fig S9: ODCs of all subjects (n = 22) are plotted for isoflurane medium concentration. Y-axis is SO_2_ in percent and x-axis is PO_2_ in mmHg.


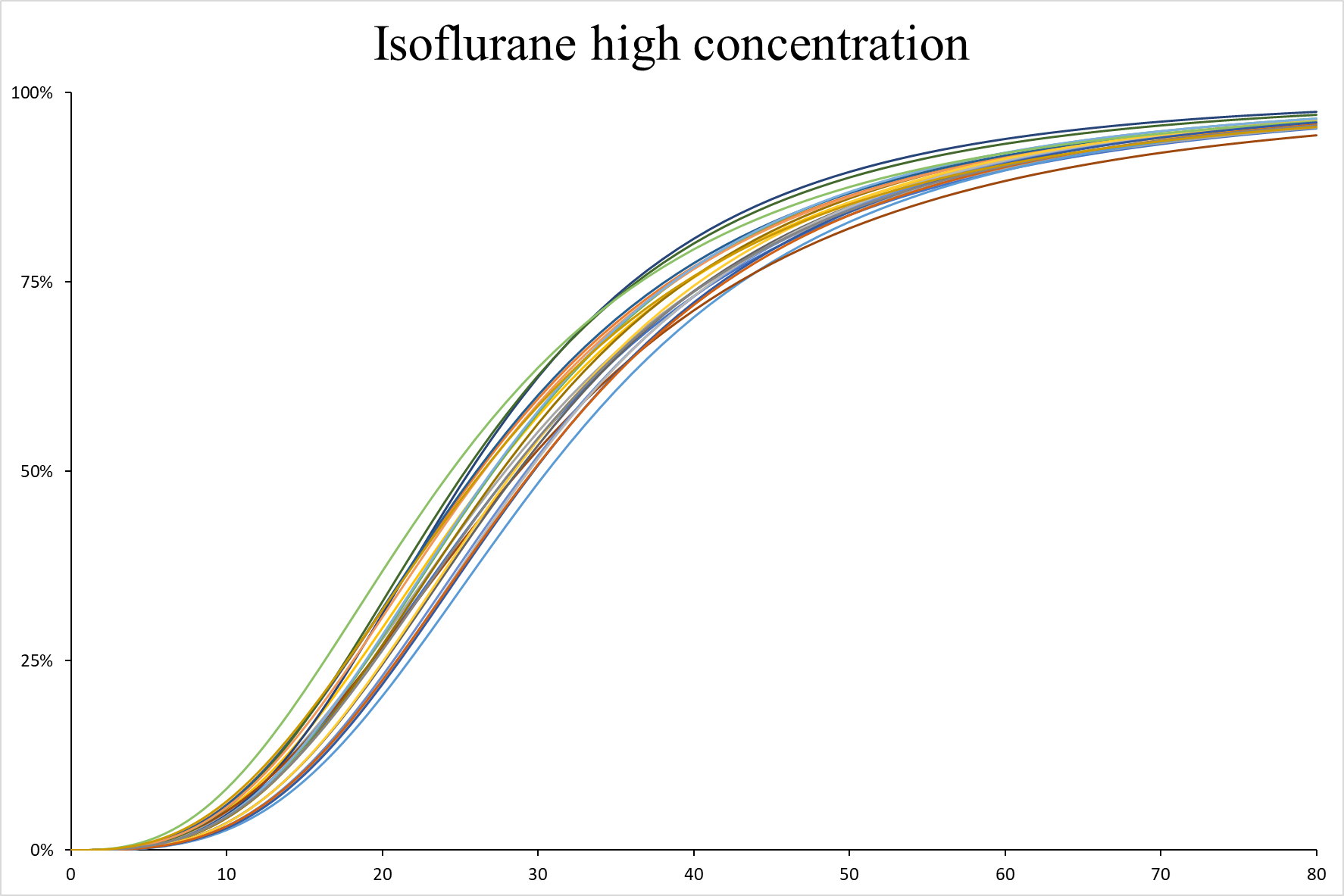


Fig S10: ODCs of all subjects (n = 22) are plotted for isoflurane high concentration. Y-axis is SO_2_ in percent and x-axis is PO_2_ in mmHg.


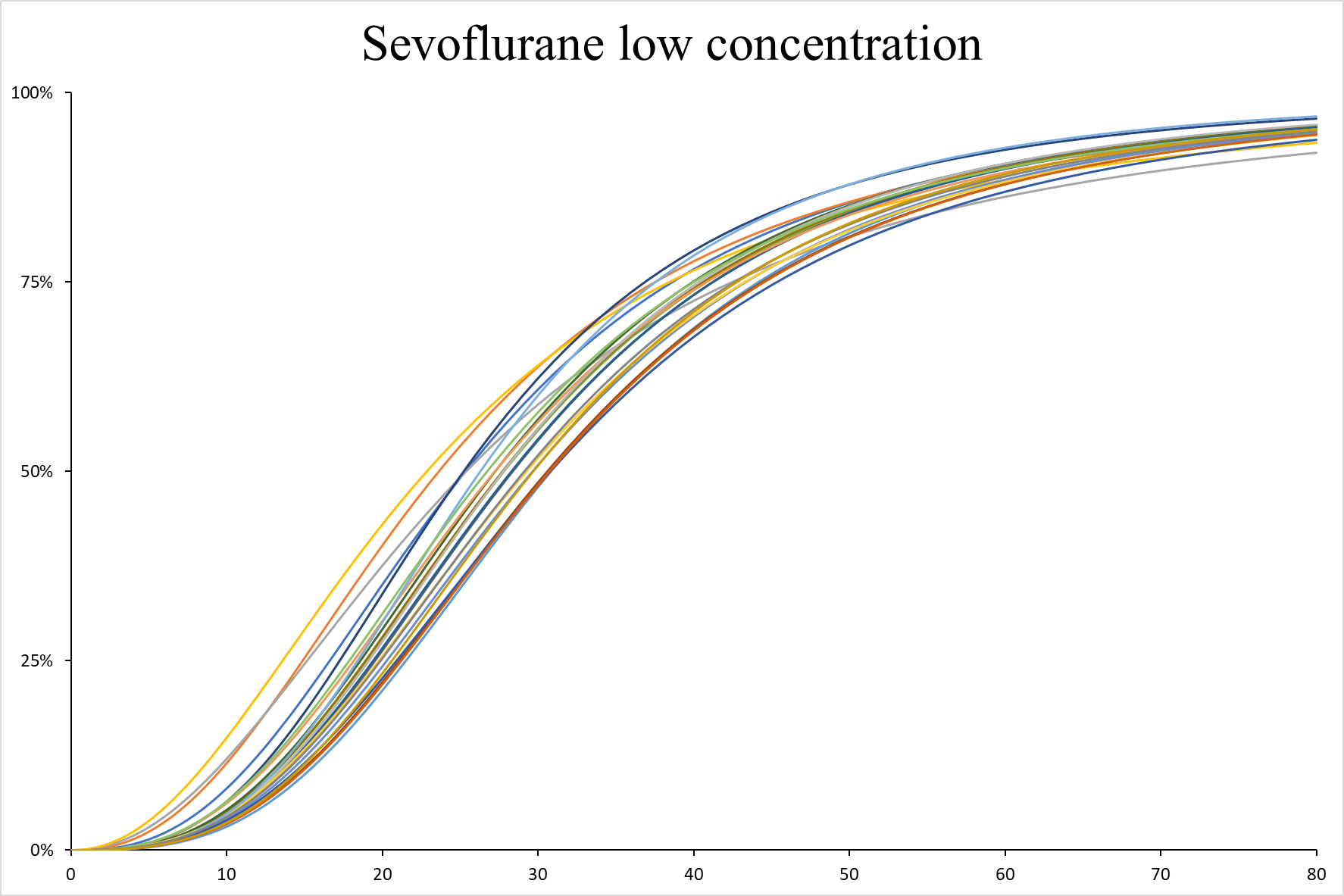


Fig S11: ODCs of all subjects (n = 22) are plotted for sevoflurane low concentration. Y-axis is SO_2_ in percent and x-axis is PO_2_ in mmHg.


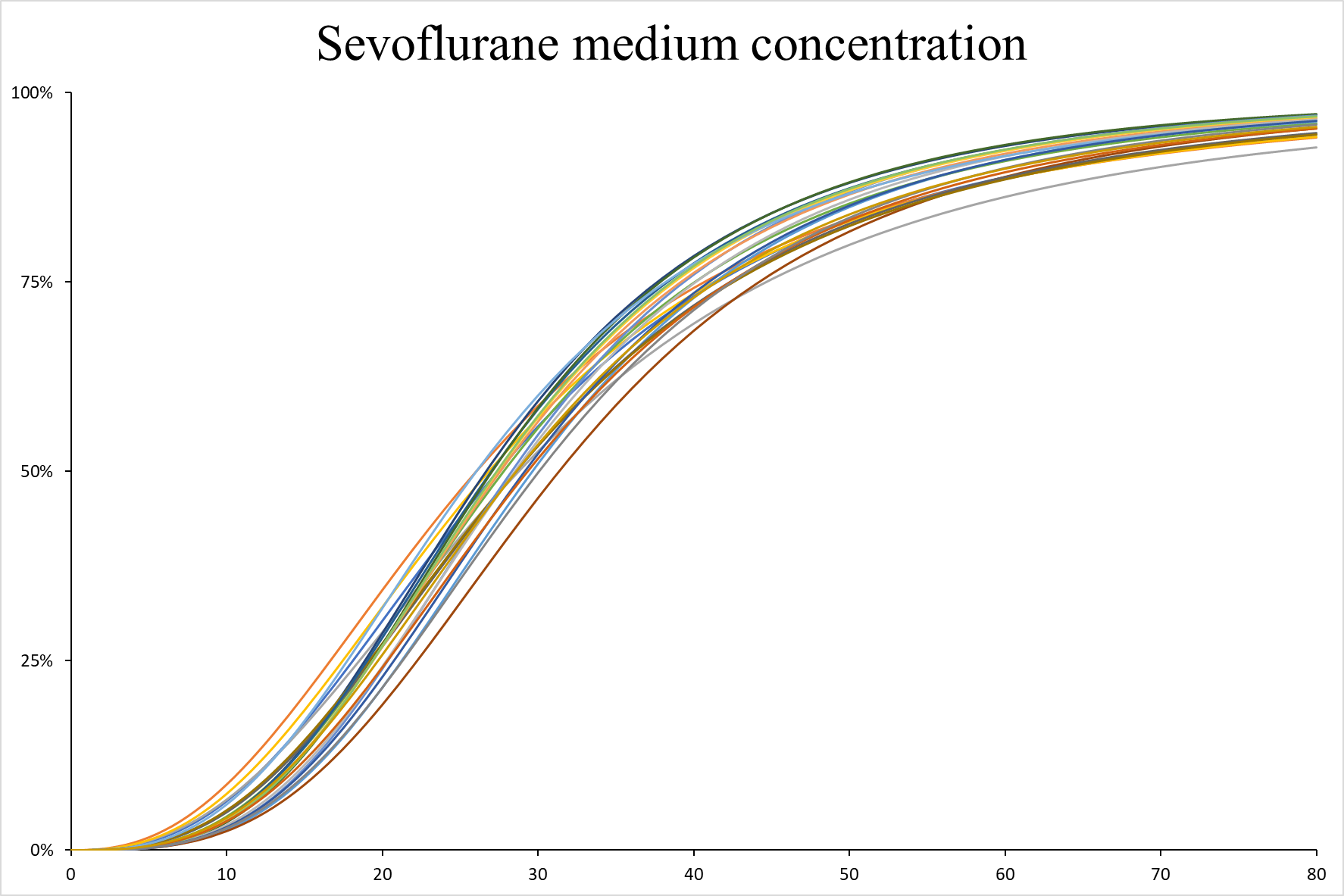


Fig S12: ODCs of all subjects (n = 22) are plotted for sevoflurane medium concentration. Y-axis is SO_2_ in percent and x-axis is PO_2_ in mmHg.


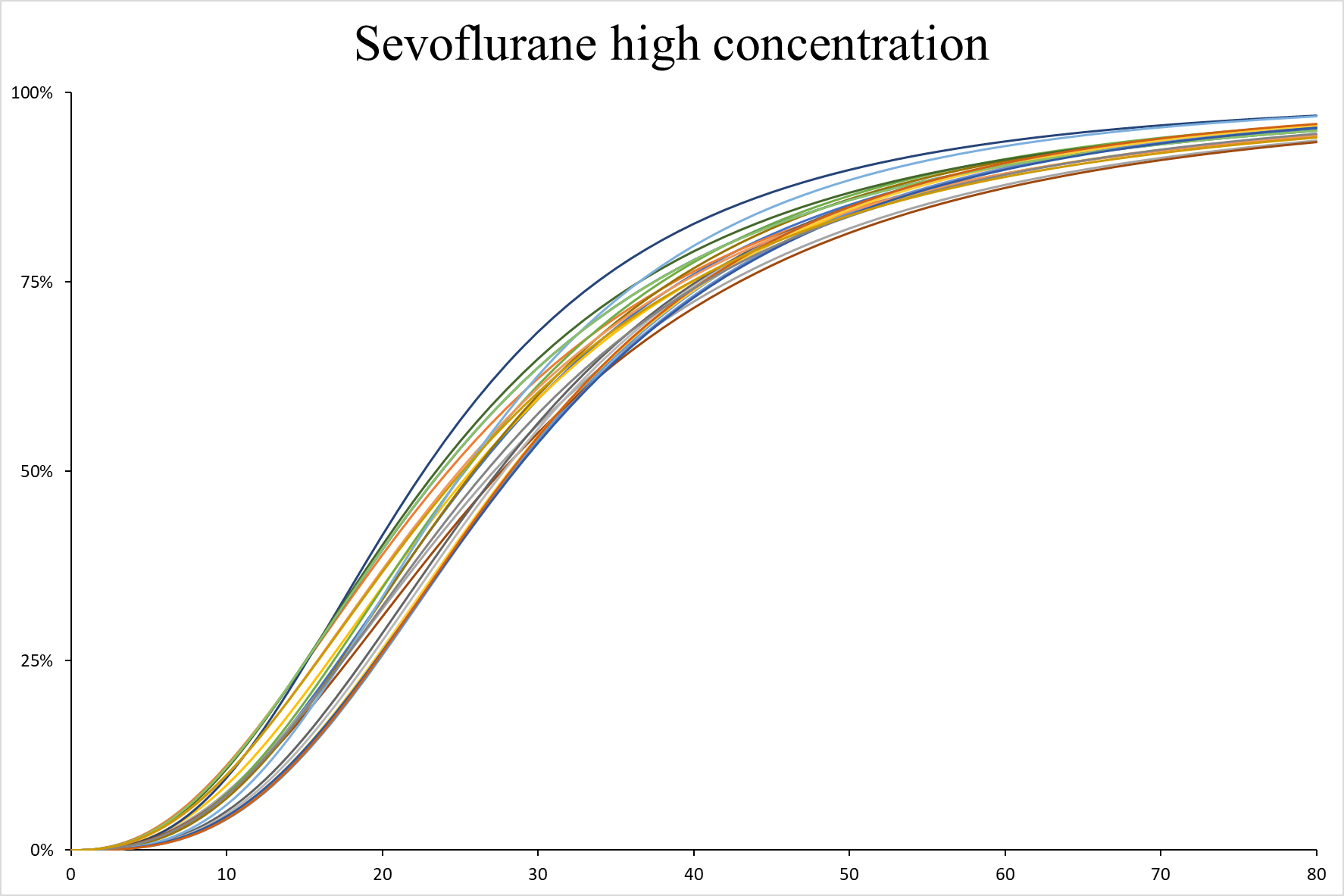


Fig S13: ODCs of all subjects (n = 22) are plotted for sevoflurane high concentration. Y-axis is SO_2_ in percent and x-axis is PO_2_ in mmHg.
